# Assessment of Linguistic Profile of Oral-Language-Proficient Hearing-Impaired Children Using Clinical Evaluation of Language Fundamentals: Fifth Edition (CELF5)

**DOI:** 10.3390/children11121458

**Published:** 2024-11-29

**Authors:** Montserrat Durán-Bouza, Lorena Pernas, Juan-Carlos Brenlla-Blanco

**Affiliations:** Department of Psychology, University of A Coruña, 15071 A Coruña, Spain; montserrat.duran.bouza@udc.es (M.D.-B.); lorena.pernas@udc.es (L.P.)

**Keywords:** language skills, CELF5, children with hard hearing

## Abstract

Background/Objectives: Specific tests for the assessment of language development and language skills in deaf children are scarce. For this reason, parent inventories and/or standardized tests that are reliable and valid in the hearing population are used. The main aim of this study was to assess the usefulness of the Clinical Evaluation of Language Fundamentals 5 (CELF5) in determining the language skills of hearing-impaired children in a comprehensive way in comparison to their hearing peers. Methods: The sample consisted of 70 deaf and 73 hearing children aged 5–11 years. Although the results show statistically significant differences in language skills between deaf and hearing children, deaf children scored around average on 8 of the 12 subtests of the CELF5. Results: Children using total communication modality had the highest scores, followed by those using oral language and sign language. The CELF5 subtests showed high internal consistency in the deaf group. A percentile scale was also developed for this population group. Conclusions: The CELF5 showed to be a reliable test for the assessment of receptive and expressive language in children with deafness who are competent in oral language. However, further research is needed to develop language assessment tests adapted to the deaf population that are sensitive to different communication modalities.

## 1. Introduction

Research indicates a scarcity of specific tests to assess language development and skills in children with deafness. Both researchers and professionals in this field commonly rely on parent inventories assessing receptive and expressive language, as well as standardized tests exhibiting high reliability and validity in hearing populations.

Regarding parent questionnaires, the MacArthur Communicative Development Inventory (CDI) [1] and the Questionnaire for Infants and Toddlers (CSBS) [2] stand out in the deaf population. The CDI evaluates language and communication in children aged 8 to 30 months, while the CSBS identifies early developmental warning signs in children aged 6 to 24 months.

As for standardized tests adapted to Spanish, the most widely used to assess language skills in deaf children are the Reynell Language Development Scale III (RDLS-III), the Preschool Language Scale in Spanish (PLS-5), the Peabody Picture Vocabulary Test (PPVT-III), and the Clinical Evaluation of Language Fundamentals, both the preschool version CELF-P2 and the version for children over 5 years of age CELF5. The RDLS-III [3] and the PLS-5 [4] enable the assessment of expressive and comprehension language in early ages (1.5 to 7 years), incorporating interactive elements such as toy use. The Peabody-III [5] focuses on receptive language in children from 30 months to adults, particularly useful for populations with expressive language difficulties. The CELF-P2 [6] and CELF5 [7] determine language competence profiles in children aged 3 to 6 years and 5 to 15 years, respectively.

In our study, we opted to utilize CELF5 to comprehensively assess language skills in children with hearing impairments, given its capacity to identify language and communication disorders. The choice of the test was also based on the data obtained in a systematic review by Vázquez Mosquera [8], regarding standardized tests for neuropsychological assessment in deaf children. Forty studies involving 62 tests were analyzed. The results indicated that the CELF5 was the most widely used test with the highest reliability in the assessment of language skills.

The clinical utility of this test battery was evaluated by its application in special populations. The results demonstrated the CELF5’s ability to discriminate between children with typical language development and those with language disorders, learning disorders, reading/writing difficulties, and autism spectrum disorder [7]. Subsequent studies corroborated its discriminative capacity. For instance, Braconnier and Siper [9] integrated the CELF5 into the neuropsychological assessment of children with autism spectrum disorder, finding it adaptable for assessing language in this population.

Recently, Veerabudren et al. [10] employed CELF5 to assess language in children with learning difficulties, observing significant differences compared to the control group, thus affirming its validity in identifying language difficulties.

Concerning hearing difficulties, the literature indicates [6,8] the use of CELF-P and/or CELF5 to evaluate language and establish linguistic profiles in children with hearing loss who use oral or total communication modalities with visual supports (bimodal, cued speech, and early reading and writing). Most of the studies tend to use tasks that assess listening comprehension and, therefore, do not require a verbal response [11,12]. The aim is to mitigate task demands and scoring ambiguities due to distorted speech. The subtests used are CELF-P2 “concepts and following directions” for children under 5 years and the CELF5 “following directions” for children aged 5 and older.

Studies using CELF subtests to analyze auditory comprehension in deaf versus hearing children highlight comparable grammatical morphology organization [13], as well as the association between executive functioning, lexical development, and language comprehension in deaf children [14].

In other studies, such as that of Malhotra et al. [15], the complete CELF-P and CELF5 batteries were used to assess the evolution of language skills in children with asymmetrical deafness after cochlear implant placement. The results showed improvements in scores on all subtests at 6-month follow-ups.

The literature review shows that earlier versions of the CELF have also been used to assess the benefits of oral therapies in the language development of children with hearing difficulties, specifically Auditory–Verbal Therapy [16,17].

In conclusion, it can be stated that when comparing the expressive and receptive language of children with deafness to that of their hearing peers, regardless of the test used, there are differences. Specifically, these differences are related to the comprehension and use of grammatical morphemes and word segmentation [18], syntactic organization [19], vocabulary learning [20], and the use of fewer co-referential and cohesive elements, as well as less discursive updating [21].

In summary, the present study aims to explore how language components such as grammar, vocabulary, and pragmatics develop in the absence of full auditory capacity. Language assessment will be conducted using the CELF-5, a standardized test employed to evaluate specific linguistic competencies, such as grammar and vocabulary, in children with hearing loss. This assessment provides data on particular areas of strength and challenge in oral language, which is especially useful for identifying differential patterns in the linguistic performance of deaf children with oral abilities compared to their hearing peers. Additionally, the study seeks to determine whether linguistic performance varies according to the communication modality employed, such as sign language, spoken language, or total communication. This is essential for understanding how different communicative modalities impact language development and how interventions can be tailored to optimize learning outcomes.

Most previous research on children with hearing loss has been conducted in English-speaking countries, primarily in contexts where access to assistive devices, such as cochlear implants, and inclusive education may differ from the Spanish context. These contextual factors influence language development, as educational and rehabilitative resources in Spanish-speaking environments may vary. Thus, understanding how language develops in Spanish-speaking children with hearing loss is essential for designing educational programs and interventions that are culturally and linguistically appropriate.

Since few studies use the CELF-5 to assess both expressive and receptive language in children with deafness who are competent in oral language, the main objective of this study is to analyze these skills in a Spanish sample. Additionally, linguistic performance will be compared with that of their hearing peers. Finally, based on the data obtained from children with hearing difficulties, information on the internal consistency of the CELF5 subtests according to age in the deaf group is presented, along with percentile scores.

## 2. Materials and Methods

A cross-sectional study was carried out in which data were collected on language skills in a group of deaf children compared to their hearing peers. The variables analyzed were sentence comprehension, linguistic concepts, word structure, and word classes, following directions, formulated sentences, recalling sentences, understanding spoken paragraphs, word definitions, sentence assembly, semantic relationships, and the pragmatics profile. In addition, data were obtained on general language performance, receptive and expressive language proficiency, content, structure, and linguistic memory.

### 2.1. Participants

The sample of the present study consisted of 143 participants, of whom 70 had hearing loss and 73 had normal hearing (control group). The mean age in the group with hearing loss was 8.47 years, SD = 1.95 and 8.48, and SD = 1.73 years in the control group (range 5–11 years, in both groups). In terms of gender, the group with hearing loss had 36 males and 34 females, with 39 normal hearing males and 34 females. All participants had a middle-to-high socioeconomic level.

The control group was recruited from a public school in A Coruña, while the sample of children with hearing loss was recruited from the otorhinolaryngology services of the University Hospitals of A Coruña, Vigo, Lugo and Santiago de Compostela, the Galician Federation of the Deaf, the Association of Parents with Hearing and Language Impaired Children of Murcia, the Turolense Association of Parents of Deaf Children, and a preferential educational center for the deaf in the Community of Madrid. Of the 70 participants with hearing loss, 24 attended a regular educational center, 29 attended a regular educational center with specific support, and 17 attended a preferential educational center for the deaf.

The inclusion criteria in both cases were to be aged between 5 and 11 years and not to have a diagnosis of a neurodevelopmental disorder.

Participants with hearing loss used 3 communicative modalities, oral language without augmentative or alternative systems (44 participants), sign language (11 participants), and a total communication system using bimodal/cued speech (15 participants).

Regarding the type of prostheses used, the most frequent were either hearing aids or cochlear implants together with hearing aids, as shown in Table 1. The mean age of fitting the prostheses in the group was 19.10 months. Considering the communicative modality, children using total communication had the earliest mean age of fitting the prosthesis (11.33 months), followed by those using oral modality (29.32 months) and those using sign language (31.91 months).

In total, 42.85% of the participants had profound bilateral sensorineural deafness, and 24.28% had severe sensorineural deafness, while the remaining 32.87% showed other types of hearing loss with varying degrees of severity between moderate and severe. Only one case had mild bilateral sensorineural hearing loss. Table 2 shows the type of hearing loss according to communicative modality.

Finally, the average age of diagnosis of deafness was 14.67 months for the whole group. Considering the communicative modality, children with sign language are the ones who received the diagnosis later, with a mean of 20.55 months, followed by those with the oral language modality with a mean of 17.41 months, and those who used the total communication modality with a mean of 2.33 months.

### 2.2. Instruments

For the collection of socio-demographic data, a questionnaire was prepared in which data on gender, age, socioeconomic status, place of origin, and languages spoken were collected. In the case of the group with hearing loss, questions about age at diagnosis of deafness, type of deafness, use of hearing aids, age of fitting, type of education, and communicative modality used were also included.

The instrument used to collect information about language skills was the Clinical Evaluation of Language Fundamentals 5 (CELF5) [7]. It is a standardized test to identify, diagnose, and follow up language and communication disorders in children and adolescents aged 5–15 years. It combines the different tests in three age ranges: 5 to 8 years, 9 to 12 years, and 13 to 15 years. It consists of 14 subtests that assess different language skills; this is morphosyntax, semantics, and pragmatics, as well as recall and the retrieval of spoken language. These subtests are classified according to age to obtain a core language score and five indices: receptive language index, expressive language index, language content index, language structure index, and language memory index (see Table 3).

Twelve of the fourteen subtests that make up the battery were administered: sentence comprehension, linguistic concepts, word structure, word classes, following directions, formulated sentences, recalling sentences, understanding spoken paragraphs, pragmatics profile, word definitions, sentence assembly, and semantic relationships. The main language score and 5 indices were also calculated: receptive language index, expressive language index, language content index, language structure index, and language memory index.

Each of the subtests for ages 5 to 8 is described below. Sentence comprehension contains 26 items measuring the ability to understand structural grammatical rules at sentence level. Linguistic concepts assess the ability to interpret directions containing basic concepts through 25 items. The word structure subtest has 33 items aimed at assessing the acquisition of the morphosyntactic rules of Spanish.

For ages 5 to 15 years, the subtests used are words classes whose purpose is to assess the semantic relationships between words and the categories to which they belong by means of 40 items. Following directions contains 33 items assessing procedural and short-term memory skills through a task of identifying figures and their position. Formulated sentences assesses the ability to orally produce complete, semantically and grammatically correct sentences of increasing length and complexity. Recalling sentences assesses the subject’s ability to listen to oral sentences of increasing length and complexity and to repeat them without changing the meaning, content, or structure of words or sentences. Understanding spoken paragraphs measures the ability to interpret factual and deductive information contained in an orally presented text. The number of texts used varies according to age. Subjects are assessed based on answers to 20 questions about the content of the texts. The pragmatics profile is a 48-item questionnaire completed by the assessor after the observation of conversational skills, information request, and non-verbal communication. The pragmatics activities checklist identifies the verbal and non-verbal behaviors that the subject displays in social interactions and that may affect their effective communication in the family or educational environment. To do this, the evaluator must choose three activities from the six proposed and observe the subject’s behavior when performing them, completing a 32-item questionnaire at the end.

For ages 9 to 15 years, the subtest word definitions, which assesses through 21 items the ability to define words by describing their characteristics and relationships with other words, are applied. Sentence assembly assesses the construction of grammatically correct and semantically meaningful sentences from words and groups of words. At least two correct sentences must be constructed using all the words presented. It consists of a total of 20 items. Finally, the semantic relationships subtest measures with 20 items the ability to understand sentences that contain a comparison, specific content or are formulated in the passive.

The core language score is obtained by adding the scalar score of three or four of the subtests (depending on age) to determine the linguistic competence of the child being assessed. Although the subtests may vary according to the age of the subject, those that are most discriminating and clinically sensitive in identifying possible language disorders are included. The receptive language index measures listening and listening comprehension skills and is obtained from the scalar score of two or three (depending on age) of the five subscales focusing on comprehension language. In the case of the expressive language index, it is obtained from the three subscales focusing on expressive language. The language content index assesses various aspects of semantic and lexical development. It is derived from three tests measuring semantic and lexical aspects. The language memory index assesses the interrelationship of meaning, structure, and memory based on three subscales assessing these aspects. Finally, the language structure index measures morphological and syntactic content through four subscales assessing these areas. The tests included in each of the indices vary according to the age of the children. The index scores range from 45 to 145.

The internal consistency of the subtests that make up the CELF5 is high, as the Crombach’s α values for the Spanish sample for each of the subtests are between 0.79 and 0.97.

### 2.3. Procedure

The study was approved by the Ethics Committee of the Galician Health Service (SERGAS) of the A Coruña-Ferrol health area (file number: 2019/475). To collect data on children with hearing loss aged between 5 and 11 years who attended the otorhinolaryngology services of the Galician university hospitals and to contact the families, the electronic medical records (IANUS program) were accessed with the authorization of the Ethics Committee of the SERGAS.

The otorhinolaryngology services of each of the Galician hospitals provided a quiet room for the appointment of the children and their families. Once the objective of the study had been explained, voluntary participation was requested by signing an informed consent form. After signing the consent form, sociodemographic data were collected, and the CELF5 was applied.

In the cases of the Galician Federation of the Deaf and the Association of Parents with Hearing and Language Impaired Children of Murcia, the Turolense Association of Parents of Deaf Children procedure was similar. First, the management of the associations was contacted. Once the application of the tests was authorized, the speech therapist of the institution chose the children with hearing difficulties according to the criteria for inclusion in the study and informed the families about the study. Once the informed consents had been signed, the interested families were summoned to apply for the tests.

The sample of children with deafness was completed by contacting a preferential educational center for the deaf in the Community of Madrid. Once authorization had been obtained from the school management, the guidance department contacted the families to explain the purpose and objectives of the study. In addition, it managed the signing of the informed consent form and scheduled the application of the tests in a classroom of the school so that it would interfere as little as possible with the classes of the children who were assessed.

In the case of normal hearing children, the data were collected in an infant and primary school in the province of A Coruña. Firstly, the school management were contacted, and an appointment was made to explain the purpose of the study. Following authorization by the management team, the school administration sent the families of the pupils aged 5 to 11 years the information about the study and the informed consent form. Once informed consent had been obtained from the families who agreed to participate voluntarily, a timetable was established to ensure that the evaluations interfered as little as possible with the dynamics of the school and of each child.

Both deaf and normal hearing children were tested individually, with sessions lasting between 45 and 60 min. The instructions for each test were delivered in sign language for children who used it as their primary means of communication. Although the evaluator was proficient in sign language, she received support and guidance from a Spanish sign language interpreter who was familiar with the test procedures. For children using oral communication aids (such as bimodal or cued speech), minimal assistance was needed, as their responses were quick and appropriate. If any difficulty arose during a subtest, bimodal support was provided. Finally, for the group of hearing-impaired children who communicated orally without aids, the instructions were given verbally.

### 2.4. Data Analysis

The data collected in the study were analyzed using the statistical package SPSS version 28. We began by checking whether there were differences in the language skills of deaf children compared to their normal hearing peers. For this purpose, a Student’s *t*-test was applied, presenting both the descriptive analysis and the effect size of the significant differences.

A descriptive analysis of the scalar scores obtained by the deaf group in each of the subtests of the CELF5 and of the composite scores obtained in the battery follows.

To test whether there are differences in the linguistic performance of participants with deafness considering the communicative modality used, a non-parametric test, namely, the Kruskall–Wallis test, was used.

To calculate the internal consistency of the CELF5 subtests and indices as a function of the age of the participants with deafness, Crombach’s α was applied.

Finally, considering also the age of the participants with deafness, two percentile scale tables were constructed for the subtests, main language score, and CELF5 indices.

## 3. Results

Firstly, to check whether there were differences between the group of deaf children and the control group in each of the linguistic variables assessed with the CELF5 battery, a Student’s *t*-test was applied. Table 4 shows the descriptive data regarding the scores of each of the groups in the 12 subtests (mean, standard deviation, and mean difference), the t-value, and the effect size.

As can be seen, the differences between the means of each group were statistically significant in 9 of the 12 subtests. The Bonferroni correction was applied, yielding an adjusted *p*-value of 0.004. When comparing the *p*-value for each of the nine subtests, it was observed that all *p*-values were below this threshold. Therefore, these results confirm the robustness of the effects. No significant differences were found between the group of deaf children and their normal hearing peers in sentence assembly, semantic relationships, and pragmatics profile. In the remaining subtests, the differences were statistically significant, with a high effect size in fiv of them: sentence comprehension, linguistic concepts, word structure, formulated sentences, and recalling sentences. The effect size was moderate in the remaining four subtests: word classes, following directions, understanding spoken paragraphs, and word definitions. The Bonferroni correction was also applied to the six indices, yielding an adjusted *p*-value of 0.008. When comparing the *p*-value of each index, it was observed that all *p*-values were below the adjusted threshold. As with the 12 subtests, these results confirm the robustness of the effects.

On the other hand, Table 5 describes the performance in each of the subtests of the group of children with deafness according to the scalar score. That is, according to the CELF5 criteria, a scalar score of 6 and below (SD < −1) would indicate below average performance; a scalar score of 7 (SD = −1) would indicate borderline performance; 8 to 12 (SD between + 1 and −1) at average; and 13 and above (SD ≥ +1) above average.

In the case of the three subtests where no statistically significant differences were found when compared to the control group (sentence assembly, semantic relationships, and pragmatics profile), more than 50% of the deaf children to whom they were administered obtained scores around or above the mean. In those differences where the effect size was larger, the percentage of children with deafness obtaining scalar scores at or above the mean was below 40% (sentence comprehension, linguistic concepts, word structure, and formulated sentences). However, in the case of the sentence construction subtest, 58.6% of children with hearing impairment obtained scalar scores around average or above.

In the case of the four subtests where the effect size was moderate (word classes, following directions, understanding spoken paragraphs, and word definitions), the percentage of children with deafness with scores around the mean or above exceeded 50% (61.42%, 51.42%, 57.14%, and 55.71%, respectively).

Regarding the composite scores calculated to obtain the main language score and the five indices, the criterion established by CELF5 is that scores between 85 and 100 would be at the mean, those ≤84 below, and those ≥116 above. Table 6 shows the scores obtained by the children with deafness according to these criteria in the core language score and the five indices.

To test whether there were differences in language performance in children with deafness depending on the communicative modality (oral language, sign language, or bimodal/cued speech), a non-parametric test was applied, namely, the Kruskall–Wallis test. The results showed significant differences in all but three subtests (sentence comprehension, sentence assembly, and pragmatics profile). Table 7 shows the results of this analysis.

**Figure 1:**
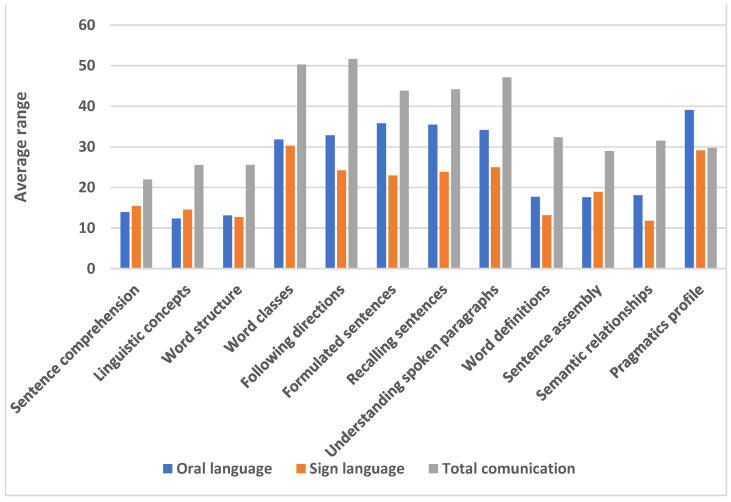
Average range obtained by hearing impaired children on the CELF5 subtests as a function of the communication modality used.

Figure 1 shows the average range assigned to each group in the Kruskal–Wallis test. This allows for the verification of which communicative modality has, on average, higher or lower values in relation to the other modalities. Children using the total communicative modality obtained the highest scores in all subtests, apart from the pragmatics profile where the scores of children using oral language were higher. The linguistic performance of children with this communicative modality follows that of those using total communication. The lowest performance corresponds to those using sign language, although in the subtests of sentence comprehension, linguistic concepts, word structure, and word classes, the differences with the oral modality are not so marked.

A similar analysis was carried out to check whether there were differences between the three communication modalities with respect to the core language score and the five CELF5 indices (see Table 8).

**Figure 2:**
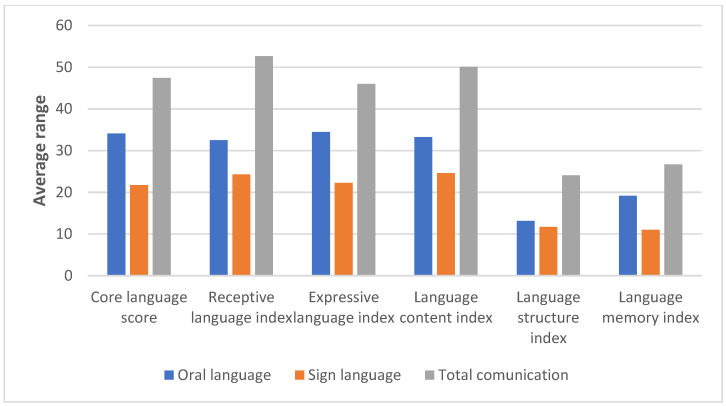
The average range obtained by children with hearing difficulties on the core language score and CELF5 index as a function of the communication modality used.

Figure 2 shows that children with full communicative modality obtain higher composite scores, followed by children who use oral language and those who use sign language.

To check the internal consistency of the CELF5 subtests and indices when assessing children with deafness, Crombach’s alpha was calculated. The calculation was made considering the age of the participants; on the one hand, it was calculated for the subtests administered to children between 5 and 8 years old and, on the other hand, for those administered to children between 9 and 11 years old.

The data obtained with deaf children aged 5–8 years show high internal consistency both for the subtests (α = 0.91 for the word structure subtest and α = 0.92 for sentence comprehension, linguistic concepts, word classes, following directions, formulated sentences, understanding spoken paragraphs, and pragmatics profile) and for the core language score and index (α = 0.90 for all of them).

The Crombach’s α values were also similar for the subtests administered to deaf children aged 9 to 11 years, as they were all 0.92 (word classes, following directions, formulated sentences, recalling sentences, understanding spoken paragraphs, word definitions, sentence assembly, semantic relationships, and pragmatics profile). As for the core language score and index, the α value was 0.90 for all of them (as above).

Finally, with the data obtained in the CELF5 in the group of children with deafness, percentile scales were drawn up to show the relative position of each subject with respect to his or her group.

In Table 9 and Table 10, for 5 and 8 years, a negative asymmetry was observed with respect to the scores obtained in sentence comprehension and word classes, indicating that only in these two subtests did most of the scores exceed the mean. This was also the case for the core language score and the receptive language and language structure indices. The kurtosis of the subtest scores was negative in most of the subtests, indicating a flat distribution with fewer negative responses than the normal curve prescribes, as in the core language score and in the four indices assessed in this age range. Only in the formulated sentence and recalling sentences subtests was a positive kurtosis obtained, indicating that some responses were repeated more than the normal curve prescribes.

However, in Table 11 and Table 12, for ages 9–11, the negative skewness in most subtests and index reveals a higher number of above-average scores. Only the asymmetry was positive in the subtests of following directions, recalling sentences and semantic relationships. As the kurtosis is negative, the distribution of scores is flat.

## 4. Discussion and Conclusions

The study’s findings reveal statistically significant disparities in the linguistic profiles assessed using CELF5 between children with hearing loss and their normal hearing counterparts. The descriptive analysis of deaf group scores indicates that in 8 of the 12 subtests, performance levels hover around or above the average. Further analysis considering communicative modalities employed (oral language, sign language, and total communication) indicates that children utilizing oral or total communication achieve higher linguistic proficiency in subtests, core language scores, and indices compared to those using sign language.

Notably, in three CELF5 subtests (sentence assembly, semantic relationships, and pragmatics profile), no statistically significant differences were observed between deaf and normal hearing children. The sentence assembly subtest evaluates the construction of grammatically and semantically accurate sentences, providing visual aids for children with hearing difficulties. Similarly, the semantic relationships subtest, measuring sentence comprehension, presents options in writing, aiding children with hearing impairments. These results echo prior research highlighting the efficacy of visual supports, whether written or manual, in enhancing language skills among children with deafness and language difficulties [9,11,12]. Noteworthy is Van Bogaert et al.’s [22] study, which asserts that deaf children lacking consistent visual supports exhibit lower lexical skills than their normal hearing peers, suggesting cochlear implants alone may not suffice for adequate speech perception. Lexical development crucially underpins linguistic comprehension and executive functioning [14].

Furthermore, in the pragmatics profile subtest, a parental questionnaire inferring ideal responses for scoring, used in studies such as Salamatmanesh et al. [23], advocates for objective tests when assessing language skills in deaf and hard-of-hearing children.

Statistical analyses unveiled larger differences between deaf and normal hearing children in subtests of sentence comprehension, linguistic concepts, word structure, formulated sentences, and recalling sentences. These tests challenge children with deafness, consistent with findings suggesting hearing secondary school students exhibit superior use and the comprehension of grammatical morphemes and word segmentation compared to deaf university students [18]. Le Normand and Thai-Van [19] note early alterations in specific syntactic components such as verbal clitics, while Fresneda and Madrid [21] observe deficits in cohesive elements among implanted deaf children.

In the linguistic concepts subtest, assessing basic concept interpretation, differences may stem from deaf children’s acquisition of less vocabulary or different vocabulary compared to normal hearing peers. This aligns with González-Cuenca et al. [20], suggesting deaf children acquire distinct semantic categories. Early intervention, as proposed by Diego-Lázaro et al. [24], may enhance vocabulary attainment, aligning developmental milestones with normal hearing peers.

In the recalling sentences test, requiring working memory and auditory attention, the results may reflect delays in executive functioning domains among children with cochlear implants [25]. Ishida and Chung [26] attribute differences in verbal working memory between deaf and normal hearing children to disparities in language processing and phonological looping abilities.

Regarding indices, significant differences were observed across all but the language memory index, possibly attributable to its auditory focus, which necessitates functional hearing absent in children with deafness until months’ post-implantation, or sometimes never acquired.

Comparing linguistic performance based on communication modality, differences emerged, with total communication users exhibiting excellent linguistic profiles like their normal hearing peers. Oral language users scored below average on several subtests and indices, primarily those reliant solely on auditory support, suggesting difficulty for children with deafness. Note that until auditory facilitation occurs, linguistic development may be delayed, lagging developmental milestones.

Sign language users attained average scores in several subtests, likely owing to visual supports or low linguistic demands, especially among those for whom sign language is a second language. Notwithstanding, disparities persisted in comparison to normal hearing peers across most tests. However, these results should be interpreted with caution, given the small sample size of those who used sign language as their communicative modality.

The study underscores high internal consistency of CELF5 among oral language-competent children with deafness, supporting its utility in assessing language skills. Percentile rankings facilitate comparison within the deaf population.

Percentile tables (Table 9, Table 10, Table 11 and Table 12) based on data obtained from deaf children are intended to provide clinicians and researchers with benchmarks for assessing language performance in this population, where standardized comparison data are often limited. By establishing specific percentiles for this group, we aim to provide tools for a more detailed understanding of language development within this population. This baseline data can facilitate more accurate identification of language strengths and weaknesses, supporting targeted interventions and contributing to improved language assessment practices for deaf children.

Limitations include small participant numbers across communicative modalities and age groups (5–8 years and 9–11 years), underscoring the need for future research to broaden and homogenize participant pools, particularly within the 12 to 15-year age bracket, for comprehensive CELF5 scale calculation. It is hoped that, in the future, group sizes will be balanced according to communication modality, considering the duration of exposure, the age of onset, and family involvement.

In conclusion, while language skills among children with deafness assessed with CELF5 trail those of normal hearing peers, disparities may hinge on communicative modality. Findings seem to suggest communication systems supporting phonology, lexicon, and oral language structure foster linguistic development in children with hearing loss. In this regard, Lara Barba et al. [27] argue that there is no consensus on the most valid assessment test based on the communicative modality used by children with deafness. They suggest that future research in this area should focus on the development of standardized instruments for language assessment in children who use sign language.

Based on these findings, CELF5 emerges as a dependable tool for receptive and expressive language assessment in oral-language-competent children with deafness. Additionally, percentile scales enable intra-group comparison.

Although early detection and intervention for deafness are advancing, ongoing efforts to refine intervention methodologies for holistic development and devise specific linguistic assessments for this population are warranted.

## Figures and Tables

**Table 1:** Type of hearing aid and communicative modality used by participants with hearing loss.

	Communicative Modality			
Type of Prosthesis	Oral Language	Sign Language	Total Communication	Total
Hearing aid	13	6	4	23
Cochlear implant	17	4	8	29
Hearing aid + cochlear implant	6	1	3	10
Osseo-integrated implant	2	0	0	2
None	6	0	0	6
Total	44	11	15	70

**Table 2:** Type of hearing loss.

	Communicative Modality			
Type of Hearing Loss	Oral Language	Sign Language	Total Communication	Total
Severe unilateral conduction deafness	3	0	0	3
Moderate bilateral conduction deafness	1	0	0	1
Severe bilateral conduction deafness	0	0	1	1
Moderate unilateral sensorineural deafness	2	0	0	2
Severe unilateral sensorineural deafness	1	0	0	1
Mild bilateral sensorineural deafness	1	0	0	1
Moderate bilateral sensorineural deafness	5	2	0	7
Severe bilateral sensorineural deafness	13	2	2	17
Bilateral profound sensorineural deafness	17	5	11	33
Moderate bilateral mixed deafness	0	0	1	1
Bilateral profound mixed deafness	0	1	0	1
Auditory neuropathy	1	1	0	2
Total	44	11	15	70

**Table 3:** CELF5 subtests and language skills assessed.

Sub-Tests	Language Skills
**Between 5 and 8 years old**	
Sentence comprehension	Processing and interpretation of structures
Linguistic concepts	Basic concepts
Word structure	Morphosyntactic rules
**Between 5 and 15 years old**	
Word classes	Relationships and semantic categories
Following directions	Understanding indications
Formulated sentences	Semantically and grammatically complete sentence
Recalling sentences	Linguistic knowledge and phonological working memory
Understanding spoken paragraphs	Cause–effect relationships, inferences, and predictions
Pragmatics profile	Conversational skills, requesting information, and non-verbal communication
Pragmatics activities checklist	Verbal and non-verbal behaviors
**Between 9 and 15 years old**	
Word definitions	Vocabulary, semantic categories, evocation of words
Sentence assembly	Syntactic knowledge, attentional skills and
Semantic relationships	memory of word order, syntagma and sentences

**Table 4:** Results of the comparison between the hearing loss group and the normal hearing group in each of the subtests and indices of the CELF5.

	Hearing Loss			Normal Hearing			Group Mean Difference			
Test/Composite Scores	Mean	Sd		Mean	Sd	*n*	Difference	t	*p*	d
Sentence comprehension	6.69	3.61		10.20	2.07	67	3.51	4.82	<0.01	1.21
Linguistic concepts	5.84	3.30		9.97	2.22	67	4.13	5.95	<0.01	1.48
Word structure	7.03	4.58		12.29	2.86	67	5.26	5.57	<0.01	1.39
Word classes	8.46	2.80		10.23	2.87	143	1.77	3.75	<0.01	0.63
Following directions	7.80	4.02		10.41	2.70	143	2.61	4.54	<0.01	0.77
Formulated sentences	9.33	5.98		13.85	4.19	143	4.52	5.21	<0.01	0.88
Recalling sentences	6.47	4.40		10.84	2.66	143	4.37	7.14	<0.01	1.21
Understanding spoken paragraphs	8.84	4.79		10.89	2.97	143	2.05	3.06	<0.01	0.52
Word definitions	11.87	4.86		14.39	2.86	76	2.52	2.76	<0.01	0.63
Sentence assembly	11.26	8.31		12.58	2.69	76	1.32	0.93	0.353	0.21
Semantic relationships	9.66	4.29		10.45	3.67	76	0.79	0.86	0.392	0.20
Pragmatics profile	8.11	3.21		9.15	3.27	143	1.04	1.91	0.058	0.32
Core language score	87.93	23.98		109.95	12.88	142	22.02	6.80	<0.01	1.15
Receptive language index	89.72	19.12		101.93	13.41	143	12.21	4.38	<0.01	0.74
Expressive language index	90.20	26.43		114.62	14.52	142	24.42	6.77	<0.01	1.15
Language content index	93.41	22.52		105.23	13.63	143	11.82	3.76	<0.01	0.64
Language structure index	76.52	17.42		106.86	13.32	66	30.34	7.87	<0.01	1.97
Language memory index	99.76	26.36		118.16	13.93	76	18.40	3.80	<0.01	0.87

**Table 5:** Scalar scores obtained by children with hearing loss on the CELF5 subtests.

Sub-Tests	Below Average (≤6)	Limit (7)	Mean (8 to 12)	Above Average (≥13)	n
Sentence comprehension	13	7	11	1	32
Linguistic concepts	19	1	12	0	32
Word structure	17	2	7	6	32
Word classes	16	11	39	4	70
Following directions	28	6	25	11	70
Formulated sentences	27	2	18	23	70
Recalling sentences	39	7	15	9	70
Understanding spoken paragraphs	24	6	23	17	70
Word definitions	23	8	35	4	38
Sentence assembly	6	4	19	9	38
Semantic relationships	9	6	13	10	38
Pragmatics profile	23	8	35	4	70

**Table 6:** Composite scores obtained by children with hearing loss on the main language score (PPL) and the 5 CELF5 index.

PPL and Index	Below Average (≤84)	Mean (85 a 115)	Above Average (≥116)	n
Core language score	28	29	12	69
Receptive language index	31	31	8	70
Expressive language index	30	24	15	69
Language content index	28	29	13	70
Language structure index	20	11	0	31
Language memory index	12	13	13	38

**Table 7:** Kruskall–Wallis test results when comparing performance on CELF5 subtests as a function of communicative modality.

CELF5 Subtests	H	*p*
Sentence comprehension	4.49	0.108
Linguistic concepts	11.95	0.003 **
Word structure	11.83	0.003 **
Word classes	10.23	0.006 **
Following directions	13.72	0.001 **
Formulated sentences	6.75	0.034 *
Recalling sentences	6.40	0.041 *
Understanding spoken paragraphs	8.08	0.018 *
Word definitions	10.16	0.006 **
Sentence assembly	5.21	0.074
Semantic relationships	9.50	0.009 **
Pragmatics profile	8.08	0.158

* *p* ≤ 0.05; ** *p* ≤ 0.01.

**Table 8:** Kruskall–Wallis test results comparing the main language score and CELF5 index as a function of communicative modality.

PPL and Index	H	*p*
Core language score	10.68	0.005 **
Receptive language index	14.98	0.001 **
Expressive language index	8.94	0.11 *
Language content index	11.43	0.003 **
Language structure index	10.10	0.006 **
Language memory index	4.86	0.088

* *p* ≤ 0.05; ** *p* ≤ 0.01.

**Table 9:** Norm table CELF5—sample with hearing loss aged between 5 and 8 years.

C	Sentence Comprehension	Linguistic Concepts	Word Structure	Word Classes	Following Directions	Formulated Sentences	Recalling Sentences	Understanding Spoken Paragraphs	Pragmatics Profile	C
99	--	--	--	--	--	--	--	--	--	99
95	≥11.70	≥11.00	≥15.35	≥11.00	≥13.00	≥12.05	≥10.05	≥13.35	≥12.35	95
90	11.00–11.69	--	13.70–15.34	--	12.00–12.99	9.70–12.04	8.70–10.04	11.70–13.34	11.00–12.34	90
80	--	--	12.40–13.69	--	9.40–11.99	9.00–9.69	7.00–8.69	9.40–11.69	10.40–10.99	80
70	9.00–10.99	9.00–9.99	11.00–12.39	10.00–10.99	8.00–9.39	8.00–8.99	5.10–6.99	8.10–9.39	10.00–10.39	70
60	7.00–8.99	6.80–8.99	7.80–10.99	9.00–9.99	7.00–7.99	6.00–7.99	4.00–5.09	7.80–8.09	8.00–9.99	60
50	--	5.50–6.79	6.00–7.79	8.00–8.99	6.00–6.99	5.00–5.99	--	7.00–7.79	7.00–7.99	50
40	6.20–6.99	4.00–5.49	4.00–5.99	7.00–7.99	5.20–5.99	4.20–4.99	3.20–3.99	6.00–6.99	6.00–6.99	40
30	5.90–6.19	3.00–3.99	--	--	4.90–5.19	2.90–4.19	2.90–3.19	5.90–5.99	5.90–5.99	30
20	2.00–5.89	--	2.00–3.99	6.00–6.99	3.00–4.89	1.00–2.89	1.60–2.89	3.00–5.89	5.00–5.89	20
10	1.00–1.99	1.30–2.99	--	5.00–5.99	2.30–2.99	--	1.00–1.59	2.00–2.99	4.00–4.99	10
5	--	1.00–1.29	1.00–1.99	4.30–4.99	1.00–2.29	--	--	1.00–1.99	3.00–3.99	5
1	--	--	--	3.00–4.29	--	--	--	--	--	1
n	32	32	32	32	32	32	32	32	32	n
Mean	6.69	5.84	7.03	8.09	6.56	5.31	4.28	6.75	7.47	Mean
Sd	3.61	3.30	4.58	2.25	3.37	3.51	2.75	3.46	2.79	Sd
Variance	13.06	10.91	21.00	5.06	11.35	12.29	7.56	11.94	7.81	Variance
Skewness	−0.27	0.14	0.43	−0.20	0.32	0.43	0.89	0.11	0.20	Skewness
Kurtosis	−0.96	−1.42	−1.12	−0.88	−0.61	0.41	0.59	−0.51	−1.03	Kurtosis

**Table 10:** Norm table CELF5—sample with hearing loss aged between 5 and 8 years.

C	Core Language Score	Receptive Language Index	Expressive Language Index	Language Content Index	Language Structure Index	C
99	--	--	--	--	--	99
95	≥102.20	≥105.75	≥108.00	≥106.10	≥102.20	95
90	100.40–102.19	101.40–105.74	103.20–107.99	104.00–106.09	100.40–102.19	90
80	94.20–100.39	98.80–101.39	96.00–103.19	98.80–103.99	94.20–100.39	80
70	87.00–94.19	96.00–98.79	87.80–95.99	94.00–98.79	87.00–94.19	70
60	85.00–86.99	86.60–95.99	80.60–87.79	86.80–93.99	84.00–86.99	60
50	80.00–84.99	81.00–86.59	76.00–80.59	78.50–86.79	80.00–83.99	50
40	71.00–79.99	78.00–80.99	66.00–75.99	74.40–78.49	71.00–79.99	40
30	64.80–70.99	73.70–77.99	62.00–65.99	72.00–74.39	65.20–70.99	30
20	57.40–64.79	69.00–73.69	55.80–61.99	68.00–71.99	59.00–65.19	20
10	46.00–57.39	60.80–68.99	47.00–55.79	59.00–67.99	49.80–58.99	10
5	40.00–45.99	57.00–60.79	45.00–46.99	58.30–58.99	45.00–49.79	5
1	--	--	--	57.00–58.29	--	1
n	31	32	31	32	31	n
Mean	75.84	82.91	75.10	81.81	76.52	Mean
Sd	18.63	14.84	19.47	15.40	17.42	Sd
Variance	349.07	220.22	379.09	237.06	303.59	Variance
Skewness	−0.37	−0.08	0.07	0.15	−0.22	Skewness
Kurtosis	−0.84	−1.07	−1.11	−1.10	−1.00	Kurtosis

**Table 11:** Norm table CELF5—sample with hearing loss aged between 9 and 11 years.

C	Word Classes	Following Directions	Formulated Sentences	Recalling Sentences	Understanding Spoken Paragraphs	Word Definitions	Sentence Assembly	Semantic Relationships	Pragmatics Profile	C
99	--	--	--	--	--	--	--	--	--	99
95	≥14.00	≥16.00	≥19.00	≥16.05	≥19.00	≥18.05	≥16.00	≥17.00	≥14.15	95
90	--	15.10–15.99	--	15.00–16.04	18.10–18.99	18.00–18.04	15.00–15.99	16.10–16.99	12.20–14.14	90
80	12.00–13.99	13.00–15.09	--	13.00–14.99	15.20–18.09	17.00–17.99	14.00–14.99	14.20–16.09	11.0012.19	80
70	10.00–11.99	12.00–12.99	17.30–18.99	11.30–12.99	13.00–15.19	15.00–16.99	12.00–13.99	12.00–14.19	--	70
60	--	9.40–11.99	15.40–17.29	10.00–11.29	12.40–12.99	14.00–14.99	11.00–11.99	11.00–11.99	10.00–10.99	60
50	9.00–9.99	8.50–9.39	13.50–15.39	8.00–9.99	11.00–12.39	12.00–13.99	10.00–10.99	9.50–10.99	9.00–9.99	50
40	8.00–8.99	8.00–8.49	11.60–13.49	7.00–7.99	10.00–10.99	11.60–11.99	9.00–9.99	7.60–9.49	8.00–8.99	40
30	7.00–7.99	6.70–7.99	9.00–11.59	5.00–6.99	7.70–9.99	10.70–11.59	8.70–8.99	7.00–7.59	7.00–7.99	30
20	5.80–6.99	4.80–6.69	7.80–8.99	3.00–4.99	5.00–7.69	7.00–10.69	7.00–8.69	6.00–6.99	5.00–6.99	20
10	4.00–5.79	3.00–4.79	4.00–7.79	1.00–2.99	3.00–4.99	3.80–6.99	4.00–6.99	3.90–5.99	3.90–4.99	10
5	2.90–3.99	1.00–2.99	1.00–3.99	--	2.90–2.99	2.00–3.79	3.90–3.99	2.90–3.89	2.00–3.89	5
1	1.00–2.89	--	--	--	1.00–2.89	--	2.00–3.89	1.00–2.89	--	1
n	38	38	38	38	38	38	38	38	38	n
Mean	8.76	8.84	12.71	8.32	10.61	11.87	10.05	9.66	8.66	Mean
Sd	3.18	4.26	5.55	4.708	5.08	4.86	3.58	4.29	3.47	Sd
Variance	10.13	18.14	30.81	22.168	25.81	23.58	12.81	18.39	12.70	Variance
Skewness	−0.30	0.00	−0.53	0.017	−0.12	−0.62	−0.26	0.09	−0.07	Skewness
Kurtosis	−0.17	−0.84	−0.81	−1.094	−0.90	−0.47	−0.49	−0.81	−0.22	Kurtosis

**Table 12:** Norm table CELF5—sample with hearing loss aged between 9 and 11 years.

C	Core Language Score	Receptive Language Index	Expressive Language Index	Language Content Index	Language Memory Index	C
99	--	--	--	--	--	99
95	≥129.30	≥129.00	≥141.20	≥137.40	≥144.10	95
90	126.00–129.29	127.00–128.99	135.00–141.19	137.00–137.39	131.70–144.09	90
80	122.20–125.99	117.00–126.99	126.00–134.99	122.00–136.99	122.40–131.69	80
70	116.60–122.19	106.20–116.99	120.60–125.99	117.20–121.99	116.60–122.39	70
60	108.80–116.59	97.80–106.19	113.60–120.59	114.00–117.19	113.00–116.59	60
50	98.00–108.79	94.00–97.79	106.00–113.59	106.00–113.99	103.00–112.99	50
40	93.00–97.99	89.80–93.99	93.00–105.99	96.80–105.99	91.00–102.99	40
30	85.00–92.99	80.00–89.79	88.40–92.99	92.40–96.79	82.70–90.99	30
20	79.00–84.99	77.40–79.99	75.80–88.39	76.80–92.39	77.00–82.69	20
10	69.20–78.99	67.00–77.39	68.60–75.79	67.70–76.79	63.40–76.99	10
5	47.90–69.19	52.85–66.99	51.75–68.59	58.45–67.69	45.00–63.39	5
1	46.00–47.89	50.00–52.84	47.00–51.74	48.00–58.44	--	1
n	38	38	38	38	38	n
Mean	98.61	94.61	102.53	102.21	99.76	Mean
Sd	22.89	21.26	25.08	23.95	26.36	Sd
Variance	523.87	451.76	629.23	573.47	695.05	Variance
Skewness	−0.47	−0.05	−0.34	−0.38	−0.27	Skewness
Kurtosis	−0.45	−0.66	−0.67	−0.57	−0.64	Kurtosis

## Data Availability

The data are not publicly available due to privacy.
